# Infertility in the Moroccan population: major risk factors encountered in the reproductive health centre in Rabat

**DOI:** 10.11604/pamj.2018.30.195.13849

**Published:** 2018-07-05

**Authors:** Amal Benbella, Siham Aboulmakarim, Houyam Hardizi, Asmaa Zaidouni, Rachid Bezad

**Affiliations:** 1 Assisted Reproductive Technology Unit of the Reproductive Health Centre, University Hospital Ibn Sina, Rabat, Morocco; 2 Laboratory of Medical Biotechnology (Med Biotech), Faculty of Medicine and Pharmacy, University Mohamed V, Rabat, Morocco

**Keywords:** Sexually transmitted infection, tuberculosis, obesity, smoking, alcohol

## Abstract

Infertility is responsible for a major cost burden for patients and health care systems. Therefore identifying preventable risk factors for infertility may contribute to the development of more cost-effective approaches to solving the infertility problem. However, such investigations have never been conducted in Morocco. Thereby, the objective of the present study was to determine the occurrence and distribution of these factors among Moroccan infertile couples. This retrospective study included 1265 infertile couples who attended the Assisted Reproductive Technology Unit of the Reproductive Health Centre of the University Hospital Ibn Sina in Rabat. All couples had been infertile for at least 1 year and both partners were fully investigated. Couples had primary and secondary infertility in 77.2% and 22.8% of cases, respectively. Women were overweight in 47.9% of cases and obese in 25.8% of cases while men were overweight in 44.6% of cases and obese in 14.7% of cases. 18.9% of women and 17.5% of men had a previous abdominopelvic or urogenital surgery. A history of sexually transmitted infection was reported by 7% of men and 5.8% of women. A history of tuberculosis was found 9.3% of women and 4.8% of men. In addition, 31.7% of men were cigarette smokers and 8.4% consumed alcohol. The study showed that preventable risk factors of infertility are common among Moroccan infertile couples. However, additional studies are required to investigate each factor and its relation to infertility in the Moroccan population.

## Introduction

Reproduction is one of the naturally desired human purposes and is indispensable for the continuation of every society [1]. However, approximately 8% of couples worldwide and 10% to 15% of those in developed countries have difficulty conceiving [2]. In many societies, especially in low and middle-income countries, infertility means not only the inability to conceive but also a failure to achieve parenthood and assure the family lineage [3]. Thereby, it exposes its sufferers to stigmatization, social isolation and loss of status [4]. In addition to the psychological stress and anxiety, infertility is also responsible for a major cost burden for the patients and the health care system [4]. Thus, it represents a common concern to public health professionals and public policy makers [4, 5]. Although assisted reproductive technology has significantly improved the chances of conception of infertile couples, this treatment is costly, burdensome and not without associated morbidity [6]. Therefore identifying preventable risk factors for infertility may contribute to the development of more cost-effective approaches to solving the infertility problem [6]. In fact, in addition to anatomical, endocrinological and genetic factors of infertility, lifestyle, diet, environmental conditions and reproductive behaviors may also impair fertility [2]. However, such investigations have never been conducted in Morocco. Thereby, the objective of the present study was to determine the occurrence and distribution of these preventable factors in the infertile population of the Assisted Reproductive Technology Unit of the Reproductive Health Centre of the University Hospital Ibn Sina in Rabat. The identification of such factors will then provide more evidence for the growing public health efforts to promote healthy lifestyles and disease prevention.

## Methods

In this retrospective study the medical records of 1265 infertile couples attending the Assisted Reproductive Technology Unit of the Reproductive Health Centre of the University Hospital Ibn Sina in Rabat, from October 2013 to March 2017, were reviewed. The complete history of all patients was detailed and a complete physical examination was conducted. Only couples who had been infertile for at least 1 year, whose investigation had been completed and of whom both partners had received a diagnosis, were admitted to the study. For men, the history of pubertal development, cryptorchidism, inguinal surgery, orchitis, testicular torsion, sexually transmitted diseases, cigarette smoking, alcohol consumption, drugs or hormones intake as well as a history of exposure to chemicals were documented. The physical examination evaluated the size and position of the testes and the presence of varicocele and any other congenital or acquired anomalies. For women, a particular attention was paid to the pattern of pubertal development, menstrual history, abdominal and pelvic surgery, cigarette smoking, alcohol consumption, drugs or hormone intake and sexually transmitted diseases. Sexual history focused on libido, erectile function, frequency and timing of intercourse. A detailed gynecologic examination was performed to ascertain congenital or acquired anomalies or diseases such as endometriosis. Specific investigations were carried out for the male partner including semen analysis and semen culture. Hormone analysis, ultrasonography and Doppler ultrasound of the scrotum were performed in some cases. Semen analysis was done according to the World Health Organization laboratory manual. For the female partner, the specific investigations performed included hormone analysis, ultrasonography and hysterosalpinography for tubal patency assessment. Hysteroscopy was performed in some cases. Laparoscopy was done when indicated to study tubal anomalies and to look for endometriosis. Statistical analysis was performed using the software package SPSS 20 (IBM) (Statistical Package for the Social Sciences). The study was approved by the Ethics Committee of Biomedical Research of the University Mohammed V of Rabat, Morocco.

## Results

This study included 1265 infertile couples. The median age of men was 39 ± 7 years (range, 21-67 years), and the median age of women was 32 ± 5.4 years (range, 18-45 years). The infertility was primary in 977 (77.2%) couples and secondary in 288 (22.8%) couples. The median duration of infertility at the time of the first medical consultation in the infertility unit was 5 ± 4 years (range, 1-24 years). The age range for menarche was between 10 and 19 years (median 13 ± 1.4 years). Table 1 gives the history of the male and the female partners. The median body mass index of women was 27.4 ± 4.5 Kg/m^2^ (range, 18-50 kg/m^2^) and the median body mass index of men was 26 ± 3.8 Kg/m^2^ (range, 19-39 Kg/m^2^). An ovulatory disorder was identified in 177 (14%) women with an overweight and in 120 (9.5%) obese women. Moreover, 136 (10.8%) overweight women and 81 (6.4%) obese women had a polycystic ovary syndrome.Among the female partners, 18.9% had previous abdominal or pelvic surgical treatments. Appendicitis, peritonitis, and intestinal occlusions were reported in 33 (2.6%), 7 (0.5%) and 2 (0.2%) cases, respectively. In 9 cases, the patients could not remember the type of surgery. Fifteen (1.2%) women who had appendectomies and all the women who were operated for peritonitis and intestinal occlusions presented a tubo-peritoneal factor. The history of surgical treatment showed that 17.5% of men had abdominal or urogenital surgery. Eighteen (1.4%) men had surgery for inguinal hernia. Twenty-six (2.1%) men had unilateral or bilateral orchiopexy for undescended testicle diagnosed at an advanced age among them 3 patients (0.2%) had unilateral orchiectomies. Unilateral or bilateral varicocele ligations were performed in 117 (9.2%) men and 2 (0.2%) patients had orchiectomies after a testicular injury.

**Table 1:** History of the study couples

	Female partner	Male partner
***Age, median ± SD***	32 ± 5.4	39 ± 7
***Body Mass Index, n (%)***		
Underweight	2 (0.2%)	0
Normal range	331 (26.2%)	515 (40.7%)
Overweight	606 (47.9%)	564 (44.6%)
Obesity	326 (25.8%)	186 (14.7%)
***Surgical treatment, n (%)***		
Uterine surgery	87 (6.9%)	-
Tubo-ovarian surgery	101 (8%)	-
Male genital surgery	-	183 (14.5%)
Digestive surgery	42 (3.3%)	38 (3%)
***Infectious disease, n (%)***		
Chlamydia Infection	39 (3.1%)	53 (4.2%)
Gonorrhea	11 (0.9%)	25 (2%)
Trichomoniasis	23 (1.8%)	11 (0.9%)
Tuberculosis	118 (9.3%)	61 (4.8%)
***Cigarette smoking, n (%)***	11 (0.9%)	401 (31.7%)
Age at starting smoking, median ± SD (range)	20 ± 3.4 (16–29)	20 ± 5.3 (10–39)
Number of cigarettes per day, median ± SD (range)	10 ± 5.5 (3–20)	18 ± 9.7 (3–50)
***Alcohol use, n (%)***	5 (0.4%)	106 (8.4%)

A history of sexually transmitted infections was found in 73 (5.8%) women and 13 (1%) of them had a pelvic inflammatory disease. A tubo-peritoneal factor was found in 44 (3.5%) women with a previous history of sexually transmitted infections, and in all patients with a previous history of pelvic inflammatory disease. On the other hand, 118 women reported a history of tuberculosis, 82 (6.5%) of these women had a tubo-peritoneal factor. For men, sexually transmitted infections were mentioned by 89 (7%) patients and 7 of these men (0.5%) had obstructive azoospermia. Furthermore, 61 men reported a history of tuberculosis and 18 (1.4%) of them had an obstructive azoospermia. Among the 288 couples with secondary infertility, 94 women had vaginal delivery, 46 had cesarean delivery, 70 had a spontaneous abortion and 37 had an ectopic pregnancy. Thirty-nine women reported that they previously had an induced abortion and 2 women had a history of molar pregnancy (Table 2). None of the women with a previous pregnancy reported postpartum or postabortal complications. The causes of infertility in the study couples were male infertility in 572 (45.2%) cases, ovulatory and endocrine disorders in 360 (28.4%) cases, tubal factor in 337 (26.6%) cases, uterine factor in 160 (12.6%) cases, endometriosis in 52 (4.1%) cases and in 192 (15.2%) cases, the cause remained unexplained. A polycystic ovary syndrome was diagnosed in 231 (18.3%) women and an obstructive azoospermia was identified in 89 (7%) men. Varicocele and cryptorchidism were found in 180 (14.3%) and 69 (5.5%) men, respectively.

**Table 2:** Outcome of previous pregnancies and female causes of infertility

Pregnancy Outcomen=288 (22.8%)	Female causes of infertility				
	Tubo peritoneal Factor n=100 (7.9%)	Uterine Factor n=41 (3.2%)	Ovulatory Disorders n=88 (7%)	Endometriosis n=11 (0.9%)	Normal n=99 (7.8%)
Vaginal Delivery 94 (7.4%)	27 (2.1%)	14 (1.1%)	30 (2.4%)	3 (0.2%)	33 (2.6%)
Cesarean Delivery 46 (3.6%)	21 (1.7%)	5 (0.4%)	15 (1.2%)	2 (0.2%)	14 (1.1%)
Spontaneous Abortion 70 (5.5%)	12 (0.9%)	15 (1.2%)	26 (2%)	6 (0.5%)	28 (2.2%)
Induced Abortion 39 (3.1%)	15 (1.2%)	3 (0.2%)	10 (0.8%)	0	15 (1.2%)
Ectopic pregnancy 37 (3%)	25 (2%)	4 (0.3%)	6 (0.5%)	0	8 (0.6%)
Molar Pregnancy 2 (0.2%)	0	0	1 (0.08%)	0	1 (0.08%)

Values are given as number (percentage of the 1265 of the study women). Some women had more than one infertility factor.

## Discussion

The last decades were characterized by various modifications affecting socio-economic and environmental conditions and leading to profound lifestyle changes [5]. In fact, societal and professional pressures were responsible for an increasing delay in childbearing contributing to the rise of diverse lifestyle and pathological perturbations [7]. Reproductive tract infections including sexually transmitted diseases represent the most important and most preventable risk factors for infertility [8, 9]. Early sexual debut, multiple sexual partners and lack of efficient prevention and treatment programs have led to the proliferation of sexually transmitted diseases [7, 8]. In the present study, self-reported history of sexually transmitted infections was found in 7% of men and 5.8% of women, which is very low compared to a study conducted in Mongolia, where previous sexually transmitted infections have been reported by 44.2% of men and 33.5% of women [9]. Moreover, a study conducted by the World Health Organization in 33 different countries, showed that in Africa, over 85% of women had an infertility diagnosis attributable to an infection compared with 33% of women worldwide [10]. Additionally, in another study from subSaharan Africa, a history of sexually transmitted infections was reported by 46% of participating men [10]. *Chlamydia trachomatis*, which is one of the leading organisms responsible for sexually transmitted diseases [11, 12], was reported in our study in 4.2% of men and 3.1% of women while in the Mongolian study, only 1.6% of infertile women and 1.2% of infertile men reported a previous chlamydia infection [9]. However, "being an obligate intracellular pathogen, the hallmark of *Chlamydia trachomatis*urogenital tract infection is chronicity and persistence", inducing silent infections [12]. In fact, up to two-thirds of infected women and 50% of infected men are asymptomatic [11, 12]. Therefore, most infections are undetected, untreated and underreported leading to the development of complications impairing fertility [4,12]. Although in women, the role of acute and chronic chlamydial infections in the genesis of fallopian tubes occlusions and pelvic-peritoneal adhesions is well established, the role of *Chlamydia trachomatis* in male infertility is still controversial [4, 11, 12]. Thus, in men, infertility might be due to inflammation and obstruction, particularly when both testicles are affected [11]. Like *Chlamydia trachomatis, Neisseria gonorrhea's* infection is highly associated with infertility [4]. Frequently asymptomatic in women, *Neisseria gonorrhea* can also cause acute pelvic inflammatory disease and subsequently irrevocable tubal damages [4]. In most men, this infection causes obvious symptoms making its diagnosis easier [13].

In the present study, *Neisseria gonorrhea* was reported by 2% of men and 0.9% of women and *Trichomonas vaginalis* was mentioned by 1.8% of women and 0.9% of men. These are low frequencies compared to the Mongolian study where Gonorrhea was mentioned in 26.3% of men and in 14.9% of women and Trichomoniasis was reported in 10.2% of women and in 9.1% of men [10]. In Women, *Trichomonas vaginalis*, which is frequently asymptomatic, has the ability to affect the upper genital tract and untreated the parasite can persist for 3 to 12 months in the genital tract [14]. However, data regarding its relation with tubal damage and pelvic inflammation are still weak [4]. Limited studies have shown that women with a previous *Trichomonas vaginalis*infection have about twice the risk for a tubal infertility [4]. On the other hand, being capable of phagocytizing other organisms and cells, *Trichomonas vaginalis* might enable the dissemination of other pathogens throughout the upper genital tract, thereby indirectly causing infertility [4]. In man, Trichomoniasis has been associated with infertility through inflammatory alterations or interference with sperm function [14]. In this study, women with a tubal infertility had a previous history of sexually transmitted infections in 13.1% of cases while according to the World Health Organization study almost 50% of the African couples and 11-15% of other patients in other parts of the world had infectious tubal disease [10]. Furthermore, all our patients who had a previous pelvic inflammatory disease had a tubal factor. This is a very high frequency compared to the literature [4]. In a Nigerian study, 52.8% of women with a history suggestive of pelvic inflammatory disease had tubal pathology [15]. The proportion of previous sexually transmitted diseases in our patients might be underestimated for two reasons. First, men and women are usually unaware of the infection due to its asymptomatic character. Second, sexual behaviors in Morocco are still to some extent a social taboo and therefore sexually transmitted diseases are denied and hidden.

In addition to sexually transmitted pathogens, other infectious diseases can also impair fertility such as genital tuberculosis whose incidence ranges from less than 1% in developed countries to almost 13% in the developing world [4, 16]. The insidious nature of genital tuberculosis allows it to persist and evolve without detection and infertility might be the first sign of the infection [4, 17]. In women, *Mycobacterium tuberculosis* affects the fallopian tubes in 92-100% of cases, the endometrium in 50% of cases and the ovaries in 10-30% of cases [16]. Thus, this pathogen not only provokes irreversible tubal obstructions, but also affects endometrial implantation and may induce ovulatory failure [16]. In men, genital tuberculosis affects mainly the epididymis rather than the testis. Less commonly, the prostate might be involved [13, 17]. This infection causes severe structural obstruction or anatomic distortion of the epididymis, vas deferens or ejaculatory duct with consequently in most cases obstructive azoospermia [17]. In our study, 20.2% of men with an obstructive azoospermia had a history of tuberculosis. In addition, the study women with a tubal infertility had a history of tuberculosis in 24.3% of cases, which is low compared to India where the reported frequency of genital tuberculosis is 41% in tubal factor infertility [16] and Iran where a study found that 81% of women with tubal factor had a history of tuberculosis [18]. Tubal infertility can also result from pregnancy-related infections and previous laparotomy [15]. In fact, up to 50% of all abdominal and pelvic surgery elicits adhesions [15]. Peri-tubal and para-ovarian adhesions can cause "follicular entrapment, reduced motility and mechanical blockage of the fallopian tubes thereby limiting oocyte transport" [15]. In a recent study performed by Famurewa investigating the connection between abdominal surgery and tubal infertility, a tubal pathology was found in all the women who have had upper abdominal surgery, surgery for ectopic pregnancy and appendectomy [15]. Similarly, our findings showed that all the women operated for peritonitis and intestinal occlusions presented a tubo-peritoneal factor. However, 45.5% of the women who had appendectomies and 67.6% of women with ectopic pregnancy had a tubal pathology. Among women who had cesarean section, 45.7% had tubal infertility. However, according to previous studies, it seems that cesarean section has no relationship with tubal pathology [15]. Concerning induced abortion, some studies have shown its association with a high risk of tubal infertility while other studies did not [15]. In our study, 38.5% of the women who had an induced abortion had a tubal infertility.

Another factor increasingly associated with the inability to conceive is obesity and overweight. In Morocco, 22% of women and 8% of men are obese [19]. This phenomenon is attributable to the shift in diet and the change to a more sedentary lifestyle [5, 19]. This study highlighted a high rate of overweight and obesity among infertile couples. Indeed, 73.7% of women and 59.3% of men had a body mass index of more than 25 kg/m^2^. The effects of overweight and obesity on women's fertility have been well established [6]. First, overweight and obese women are more likely to have ovulatory disorders [6]. A case-control study conducted at Harvard University comparing 2,527 infertile anovulatory women and 46,718 control subjects has shown an association between body mass index at the age of 18 years old and the risk of subsequent anovulatory infertility. In addition, weight loss in obese anovulatory women was associated to a significant improvement in ovulatory function and pregnancy rates. In the present study, women with a polycystic ovary syndrome were overweight in 58.9% of cases and obese in 35% of cases. However, not all women with polycystic ovary syndrome are obese and not all obese women suffer from polycystic ovary syndrome. Indeed, our findings showed that 22.4% of overweight women and 24.8% of obese women had a polycystic ovary syndrome. However, even ovulatory obese women experience difficulty conceiving. Indeed, a study conducted by Van der Steeg et al has reported a decrease of 4% per kg/m^2^ of the probability of spontaneous conception in women with a body mass index >29 kg/m^2^. Secondly, obesity has a negative impact on assisted reproductive technologies outcomes [20]. Studies have shown that obese women require higher doses of gonadotrophins and longer duration of ovarian stimulation [6, 20]. Cycle cancellation for inadequate response was more frequent, and the number of oocytes retrieved was lower. There was no evidence of an effect on embryo quality [20]. However, pregnancy rates were lower in obese women [6, 20]. Thirdly, obesity is associated with a high risk of pregnancy loss [20]. Indeed, Studies investigating the outcomes of pregnancies achieved with assisted reproductive technologies reported an increasing risk of pregnancy loss in overweight and obese women compared with normal weight women [20]. In order to decrease all these negative effects, all patients treated in the assisted reproductive center with a body mass index of more than 25 kg/m^2^ are enrolled in a weight loss program. In men, although high body mass index has been identified by large population studies as an independent risk factor for infertility, its relationship with sperm quality remains controversial. In fact, some studies established that there was a strong correlation between obesity and sperm parameters while other studies found no significant correlation [6, 21]. On the other hand, most studies have reported a statistically significant negative correlation between body mass index and total testosterone, free testosterone and sex hormone binding globulin [6].

In addition, it was demonstrated that overweight and obesity have a statistically significant positive correlations with DNA fragmentation index [6]. This indicates that high body mass index can affect the sperm quality independently of sperm count or other sperm parameters [6]. Other negative impacts of high body fat on male fertility are an increased seminal oxidative stress and a high proportion of erectile dysfunction [6, 21]. Studies have shown that smoking had a detrimental impact on male and female fertility [2]. According to the World Health Organisation 2008 report, the current prevalence of smoking among Moroccan adults is 14.2%, with a rate of 29.5% for males and 0.3% for females [22]. In this study, 31.7% of men were smokers while in Bayasgalan's study 48.9% of infertile men were smokers. Many studies investigating the role of cigarette smoking in the reduction of semen quality found a negative relationship between smoking and semen parameters while other studies did not observe any impact [23, 24]. However, despite the lack of decisive evidence, "the latest American Society for Reproductive Medicine Committee opinion stated that smoking can affect basic semen parameters in a dose-dependent manner". Smoking has also a negative impact on sperm function particularly its fertilization abilities. This means that even in the presence of normal semen parameters, sperm functions could still be impaired in smokers [23]. In addition, cigarettes are responsible for increased seminal oxidative stress [24], reduced vesicular and prostatic parameters and increased risk of erectile dysfunction. With regard to male sex hormones, the results of various studies were conflicting [23]. In women, cigarette smoking has been shown to negatively affect folliculogenesis and ovarian reserve. Thus, it is related to earlier menopause, menstrual disorders, and difficulty conceiving. Studies have reported that smoking women have a reduced menopausal transition by 1 to 1.5 years leading to early menopause [24]. Moreover, smoking is associated with an increased risk of assisted reproductive treatment failure, as well as pregnancy loss [4]. Indeed, increased doses of gonadotrophines are required in smoking women under ovarian stimulation for assisted reproductive treatments [24]. Frequently associated with smoking, chronic alcoholism has a deleterious impact on male and female fertility. In fact, in women, heavy alcohol consumption has been shown to be associated with a decline in ovarian function; and therefore, is responsible for infertility and reduced in vitro fertilization cycle success [25]. In the present study, only men consumed alcohol in 8.4% of cases this frequency is low compared to other countries. In the Mongolian study, 14.9% of men reported alcohol consumption. In men with chronic alcohol intake, male reproductive hormones are altered, semen quality is impaired and sexual disorders are frequent with a prevalence ranging from 8% to 58% [26].

## Conclusion

Sexually transmitted diseases, tuberculosis, obesity, cigarette smoking, alcohol consumption are all preventable infertility risk factors. This study showed that these factors are common among infertile couples. However, additional studies are required to investigate each factor and its relation to infertility in the Moroccan population. On the other hand, the study findings highlighted the possibility and necessity of preventive measures to solve the infertility problem before having recourse to expensive assisted reproductive treatments. Information, education and communication programs are cheaper; promote safer sexual behaviors and contraception; and enhance awareness of obesity and cigarette smoking health consequences.

### What is known about this topic

- Sexually transmitted diseases represent the most important and most preventable risk factors for infertility;
- The incidence of tuberculosis ranges from less than 1% in developed countries to almost 13% in the developing world;
- Obesity and overweight are a global and major concern to public health.

### What this study adds

- All the study women with a previous pelvic inflammatory disease had a tuboperitoneal infertility;
- There is a high frequency of overweight and obesity among the study population;
- The study women with a tubo-peritoneal factor had a history of tuberculosis in 24.3% of cases.

## Competing interests

The authors declare no competing interest.
